# Automatic quantification of *in vitro* NET formation

**DOI:** 10.3389/fimmu.2012.00413

**Published:** 2013-01-09

**Authors:** Volker Brinkmann, Christian Goosmann, Lars I. Kühn, Arturo Zychlinsky

**Affiliations:** ^1^Microscopy Core Facility, Max Planck Institute for Infection BiologyBerlin, Germany; ^2^Department of Cellular Microbiology, Max Planck Institute for Infection BiologyBerlin, Germany

**Keywords:** NEtosis, chromatin, immunofluorescence, segmentation, quantification

## Abstract

Neutrophil Extracellular Traps (NETs) consist of decondensed chromatin studded with granular and some cytoplasmic proteins. They are formed by activated neutrophil granulocytes, also called polymorphonuclear leukocytes (PMN) as the result of an active cell death program, named NETosis. NETosis can be induced by a wide range of stimuli including coculture of neutrophils with pathogens (bacteria, fungi, parasites, virus particles), activated platelets, or pathogen components. The first step of the NETotic cascade is stimulation of one or several receptors followed by activation of the Raf/MEK/ERK pathway that culminates in the assembly of the multimeric NADPH oxidase complex and the production of reactive oxygen species (ROS). Later, intracellular membranes disintegrate, the granular protein Neutrophil Elastase enters the nucleus and processes core histones that also get hypercitrullinated. This leads to decondensation and mobilization of chromatin. The amount of NET formation varies with the degree of stimulation, and this is dependent on the type and concentration of the stimulus. NETs can be quantified using various methods including fluorescence microscopy or measuring DNA release. Each of these methods have specific drawbacks: analysis of fluorescence microscopy is prone to subjective variations, and DNA release does not differentiate between DNA that has been released by NETosis or by other forms of cell death. Here we present a protocol to semi-automatically quantify NET formation. It relies on the observation that anti-chromatin antibodies bind more readily to decondensed chromatin present in the nuclei of cells undergoing NETosis and in the NETs. Relating the fluorescence signals of the anti-chromatin antibody to the signals of a DNA-binding dye allows the automatic calculation of the percentage of netting neutrophils. This method does not require sophisticated microscopic equipment, and the images are quantified with a public-domain software package.

## Introduction

NETs are a fibrous structure consisting of a chromatin backbone with attached globular domains (Brinkmann et al., 2004; Urban et al., 2009). These domains contain granular proteins and peptides as well as some cytoplasmic components (Urban et al., 2009). The main components of NETs are the core histones (H2A, H2B, H3, H4) which together account for about 70% of the protein mass (Urban et al., 2009). Histones have long been recognized as potent antimicrobials (Miller et al., 1942; Hirsch, 1958), and together with the microbicidal granular enzymes and peptides, NETs have been shown to have activity against bacteria, fungi, parasites, and viruses (Brinkmann et al., 2004; Beiter et al., 2006; Urban et al., 2006; Bianchi et al., 2009; Aulik et al., 2010; Behrendt et al., 2010; Abdallah et al., 2012; Saitoh et al., 2012). The intact structure is indispensable for the microbicidal activity of NETs since it ensures a high local concentration of the active components in close vicinity to the pathogens. Treatment of NETs with DNase destroys their antimicrobial potency (Brinkmann et al., 2004). Interestingly, bacteria evolved extracellular nucleases that free them from NETs and allow spreading (Beiter et al., 2006; Buchanan et al., 2006). Besides their role in host defense, NETs have been shown to be involved in blood clotting (Fuchs et al., 2010; Massberg et al., 2010; von Brühl et al., 2012) and in the activation of dendritic cells (Lande et al., 2011).

Both the generation and the destruction of NETs has to be tightly regulated to provide prompt defense against invading pathogens as well as timely coagulation and to avoid negative effects that are associated with overshooting release or reduced clearance of NETs. Lack of NET formation has severe consequences as exemplified by patients suffering from Chronic Granulomatous Disease. These patients cannot make NETs due to an inactive NADPH oxidase complex (Fuchs et al., 2007; Bianchi et al., 2009) and suffer from severe recurrent infections. On the other hand, abundance of NETs due to imbalanced production and destruction can lead to endothelial damage (Saffarzadeh et al., 2012), uncontrolled thrombus formation or induction of autoantibodies against NET components (Hakkim et al., 2010) as well as other disorders (reviewed in Kaplan and Radic, 2012) and (Brinkmann and Zychlinsky, 2012). Thus, molecules that affect the balance of NET creation and destruction can be of therapeutical relevance.

The signaling pathways that lead to NET formation are just being unraveled, many aspects so far remain obscure. Numerous compounds that either promote or prevent NET formation have been tested *in vitro*, and several read out systems have been used to estimate the amount of NET release. The results obtained in different laboratories are hard to compare since different methodologies were used and in many reports the readout was prone to individual bias. Therefore, there is a need for a method that allows automatic quantification of NET formation, which is ideally also useful for high-content screening (HCS).

We present here a protocol that uses dual channel fluorescence staining and automatic image segmentation to determine the percentage of NETotic neutrophils. An antibody against a subnucleosomal complex (Losman et al., 1992) detects relaxed chromatin which is a hallmark of NETosis (Fuchs et al., 2007) and stains both neutrophils that undergo all phases of NETosis as well as NETs after their release from dying cells (Ermert et al., 2009; Brinkmann and Zychlinsky, 2012). The DNA-intercalating dye Hoechst 33342 is used to determine the number of cells per field of view. This method does not demand a sophisticated optical setup, image analysis is performed with the public domain software ImageJ (Schneider et al., 2012). The protocol could be useful to provide unbiased results that are adequately standardized to allow comparison of data sets that were generated in different laboratories. It can also be adapted to HCS.

## Materials and methods

### PMN isolation

PMN were isolated from freshly drawn blood of healthy donors as described previously (Aga et al., 2002). Briefly, whole blood was separated by spinning on a Histopaque 1119 cushion. The resulting neutrophil-rich fraction was washed and layered on a discontinuous Percoll gradient (85 – 65% in PBS) for centrifugation. Bands on the 80, 75, and 70% layers were collected, pooled, and washed with PBS. The cells were counted and kept in PBS for the induction experiment.

### NET induction

PMN were seeded on 13 mm glass coverslips in 24-well-plates in 490 μl of RPMI supplemented with glutamine, pyruvate, and 0.5% human serum albumin at a density of 10^5^ cells per well. The plates were incubated for 15–30′ at 37 °C to allow adhesion of the cells. Prediluted aliquots of the test compounds were prepared at 50×, 5×, and 0.5× of the planned maximum concentrations. Proinflammatory cytokines were used at following concentrations: TNF-α, 1 ng/ml; G-CSF, 1 ng/ml; IL-1β, 10 ng/ml. As positive control, we used phorbol 12-myristate 13-acetate (PMA, 50 nM). Negative controls were DMSO (diluted to match the maximum dose to come with the test compounds) and seeding medium. To start the induction, 10 μl of the prepared aliquots were added to each well and the plates were kept at 37°C for 10′, 2 h (controls) or 6 h (controls and test compounds) before fixation by adding 167 μl of 8% PFA in PBS to reach a final PFA concentration of 2%. For initial analysis of the staining patterns, PMN samples prepared as described here were stimulated with PMA for various periods of time (240′, 130′, 80′, 10′ and 0′) before fixing all samples simultaneously.

For coculturing experiments, *Pseudomonas aeruginosa* strain PA14 at MOI 1, 10, and 100 was added to PMN on coverslips in 24-well plates and centrifuged (5 min at 300 × g). The plates were incubated for 5 h at 37°C.

### Sample staining

Coverslips with the fixed cells were removed from the plates and processed by floating on drops kept on hydrophobic laboratory film. After washing with PBS, the samples were permeabilized for 1′ with 0.5% Triton X100 in PBS, washed again with PBS and blocked for 20′ with blocking buffer (3% normal donkey serum, 3% cold water fish gelatin, 1% BSA, and 0.05% Tween 20 in PBS). Solutions of antibody (mouse mAB PL2-3) directed against the subnucleosomal complex of Histone 2A, Histone 2B, and chromatin (Losman et al., 1992) and against neutrophil elastase (rabbit pAB Calbiochem 481001) were applied in blocking buffer for 1–2 h. After washing with PBS, dye-conjugated secondary antibody solutions were applied for 30′–1 h (donkey anti mouse Cy3, donkey anti rabbit AlexaFluor 488, Jackson). The samples were washed with PBS and distilled water, stained with aqueous Hoechst 33342 (100 ng/ml, Sigma), washed again in distilled water and mounted with Mowiol.

### Image acquisition and batch quantification

Five images taken randomly from different regions of each coverslip in an experiment were taken with the 10× lens on a Leica DMR upright fluorescence microscope equipped with a Jenoptic B/W digital microscope camera. Exposure times of each channel were kept constant over the whole series in an experiment after calibrating on a bright representative sample to avoid saturated pixels. The image files were loaded as separate image stacks for each channel in ImageJ/FIJI software (Schindelin et al., 2012; Schneider et al., 2012). The parameters used for segmentation were controlled by scrolling through and checking the image stacks. To collect the data of total cell number, the Hoechst 33342 fluorescence image stack was binarized with Bernsen automatic local threshold function set to diameter 15 and threshold 35. Automatic particle analysis was set to 20 pixels minimum size and summarized result output. The resulting list of results was saved for further processing. We used the stack of images in the channel with the anti-chromatin immunolabeling to collect NET data. The threshold was set interactively to the minimum threshold that rendered no objects larger than 75 pixels on an image with 10′ PMA activated cells (except spontaneous NETs that are >75pixels). The result of particle analysis with a size-cut-off of 75 pixels was exported as before. Result tables of the Hoechst 33342 cell count and immunofluorescent NETs count were imported into a spreadsheet program and analyzed further. NET-rate was calculated as follows:

NET−rate [%] =100×Objects counted (chromatin channel)/Objects counted (Hoechst channel).

The standard deviation was determined by comparing the results of the five individual images analyzed for each specimen.

RGB-merged stacks of the three channels DNA, (relaxed) chromatin, and Neutrophil Elastase with an overlay of the segmented object outlines were generated and used solely as control images to visually assess and exclude the influence of non-neutrophil cells or imaging artifacts on the results. Immunostaining of Neutrophil Elastase was not further included in the analysis.

### Analysis of staining patterns

Images of the time course of PMA-activated neutrophils were taken with a 100× lens. The Hoechst 33342, Cy3, and AlexaFluor 488 images were merged into RGB with ImageJ/FIJI software, leaving the single channels open. Line selections of representative cells were made in the merged RBG image and profile plots of the same selection taken in the respective single channel images. The data lists of the profile plots were imported into a spreadsheet program and represented in combined line diagrams. For the time-course diagram, representative areas of the stained cells in the time-course were taken with the 40× lens and the images of the Hoechst DNA and the Chromatin IF channels loaded as stacks into ImageJ. Segmentation in the Cy3 (chromatin) channel with automatic threshold (“Moments”-algorithm) identified objects (nuclei … NETs) that were loaded into the ROI-manager. The intensities of the Hoechst and the Cy3 channel were measured for each ROI, the data lists were exported to a spreadsheet program and represented in a line diagram.

## Results

### Change of DNA/chromatin staining patterns during NETosis

**Figure 1:**
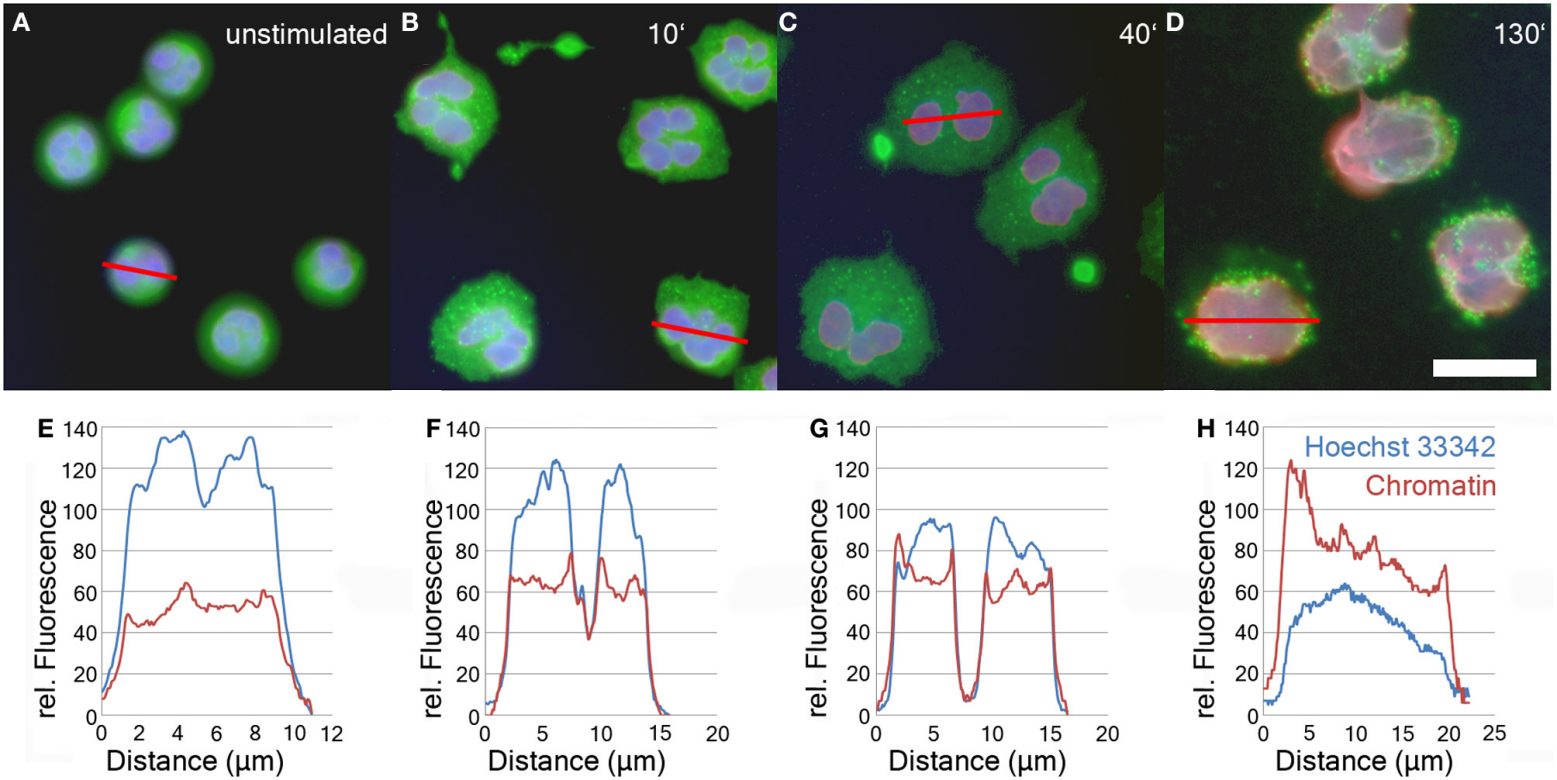
**Immunofluorescence analysis of unstimulated neutrophils (A) and neutrophils stimulated with 50 nM PMA for 10'(B), 40'(C), and 130' (D).** Blue: DNA (Hoechst 33342), red: chromatin (antibody PL2-3), green: Neutrophil Elastase. Bar represents 20 μm. Panels **1E–H** show line plot quantifications of DNA and chromatin fluorescence intensity of individual cells depicted in panels **1A–D**.

After induction of NETosis, neutrophils undergo a series of morphological modifications. The most prominent is the transformation of the nucleus which is characterized by a gradual decondensation (Fuchs et al., 2007). While unstimulated neutrophils retain a lobulated nucleus (Figure 1A), after treatment with PMA or other stimuli, neutrophils rapidly become adherent (Figure 1B). Later, their nuclei start to decondense and to lose their lobulation (Figure 1C). In the final phase of NETosis, the entire cell is filled with a homogenous mass of intermingled cytoplasm and karyoplasm (Figure 1D and Fuchs et al., 2007).

The relaxation of chromatin during NETosis results in the exposure of the epitopes of anti-chromatin antibodies and consequently a more intense fluorescence signal (Ermert et al., 2009; Brinkmann and Zychlinsky, 2012). In contrast, the staining intensity of DNA-intercalating dyes like Hoechst 33342 decreases during NETosis due to the drop in DNA concentration.

The relative staining intensity of Hoechst 33342 (blue) and the anti-chromatin antibody (red) is specified in line plots of individual cells after various periods of stimulation in Figures 1E–H. Unstimulated cells are characterized by a condensed lobulated nucleus which stains brightly with Hoechst 33342, but weakly with the chromatin antibodies (Figure 1E). This proportion of intensity values is inverted during later phases of NETosis when the nuclei decondense and allow better antibody penetration of the chromatin (Figures 1F–H). In late phases of NETosis, chromatin staining is more intense than staining with DNA-intercalating dyes resulting in a more reddish signal of the overlay of fluorescence channels (Figure 1D). In parallel, the average diameter of the DNA/chromatin staining increases from about 10 μm for the spherical unstimulated cells (Figures 1A,E) to more than 20 μm for neutrophils in late stages of NETosis (Figures 1D,H).

### Time course of PMA stimulation

**Figure 2:**
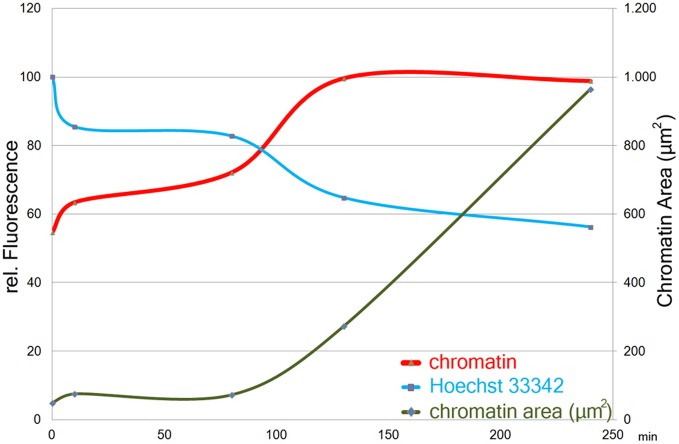
**Size (right y-axis) and staining intensities (left y-axis) of automatically segmented objects in images of chromatin- and Hoechst33342- stained cells from a time course of PMA induced NETosis.** One image with 47 objects (avg.) per timepoint was measured. Staining intensity plotted as means per timepoint of the maximum intensity-value in each object.

About 90 min after stimulation with PMA, neutrophil nuclei start to decondense. This leads to a significant increase in the area stained by the anti-chromatin antibody (Figure 2, green line). In parallel, already after 10 min of PMA stimulation, the intensity of Hoechst 33342 staining decreases (Figure 2, blue line), while the staining with anti-chromatin antibodies becomes brighter, reaching a plateau after about 120 min of stimulation (Figure 2, red line). Both Hoechst 33342 and chromatin staining intensities remain stable until most cells have reached late phases of NETosis or have released NETs after 240 min of stimulation.

### Segmentation of hoechst 33342 and chromatin immunofluorescence signals

**Figure 3:**
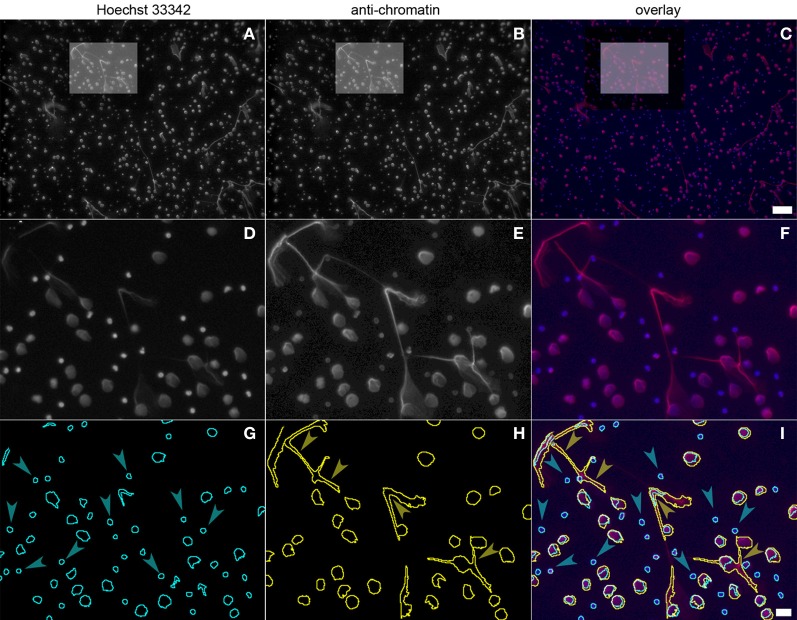
**Neutrophils stimulated with PMA for 130 min and stained with Hoechst 33342 (panel 3A, boxed area panel 3D) and PL2-3 (panel 3B, boxed area panel 3E).** The overlap is shown in panel **3C** (blue, DNA; red, chromatin; boxed area in panel **3F**). Automatic segmentation of the fluorescence signals is shown in panel **3G** (blue, DNA) and panel **3H** (yellow, chromatin). The overlay of the segmentations and the fluorescence signals is depicted in panel **3I**. Bars represent 100 μm for **(A–C)** and 20 μm for **(D–I)**.

Using 5 image sets taken with a 10× lens, about 5%, of the specimen area is recorded and at a seeding density of 10^5^ cells per well, about 1000–5000 cells are analyzed. Figure 3 depicts a sample of neutrophils stimulated with PMA for 130 min. The upper row (Figures 3A–C) displays the entire 10× micrograph, the boxed insert is magnified to better show the fluorescence signals (Figures 3D–F) and the segmentation (Figures 3G–I). The cell number per field of view is determined using the Hoechst 33342 signal (Figures 3A,D; blue in Figures 3C,F). After 130 min of PMA stimulation, a fraction of cells have decondensed nuclei and stain with the chromatin antibody (Figures 3B,E; red in **C,F**), indicating NETosis. The image stacks are automatically segmented by the ImageJ software as shown in Figure 3G (Hoechst 3342), Figure 3H (chromatin) and Figure 3I (overlay of segmentations and fluorescence signals). Cells that have not (yet) responded to the PMA stimulus are only detected in the Hoechst 33342 segmentation (blue arrowheads in Figure 3I), while NETotic neutrophils and NETs are identified in the chromatin segmentation (Figure 3H). While neutrophils even in late phases of NETosis are properly segmented, NETs that are produced by several cells are possibly counted as single particles (yellow arrowheads in Figure 3I). If this is the case, the NETosis percentage will be underrepresented.

### Measuring NET induction

To test whether the protocol is generally applicable for measuring NET induction, we tested neutrophils from different donors as well as various inducers of NET formation. These included PMA, ligands of Toll-like receptors (TLR), coculture with pathogens, (in-) organic particles as well as cytokines.

### Donor variation

**Figure 4:**
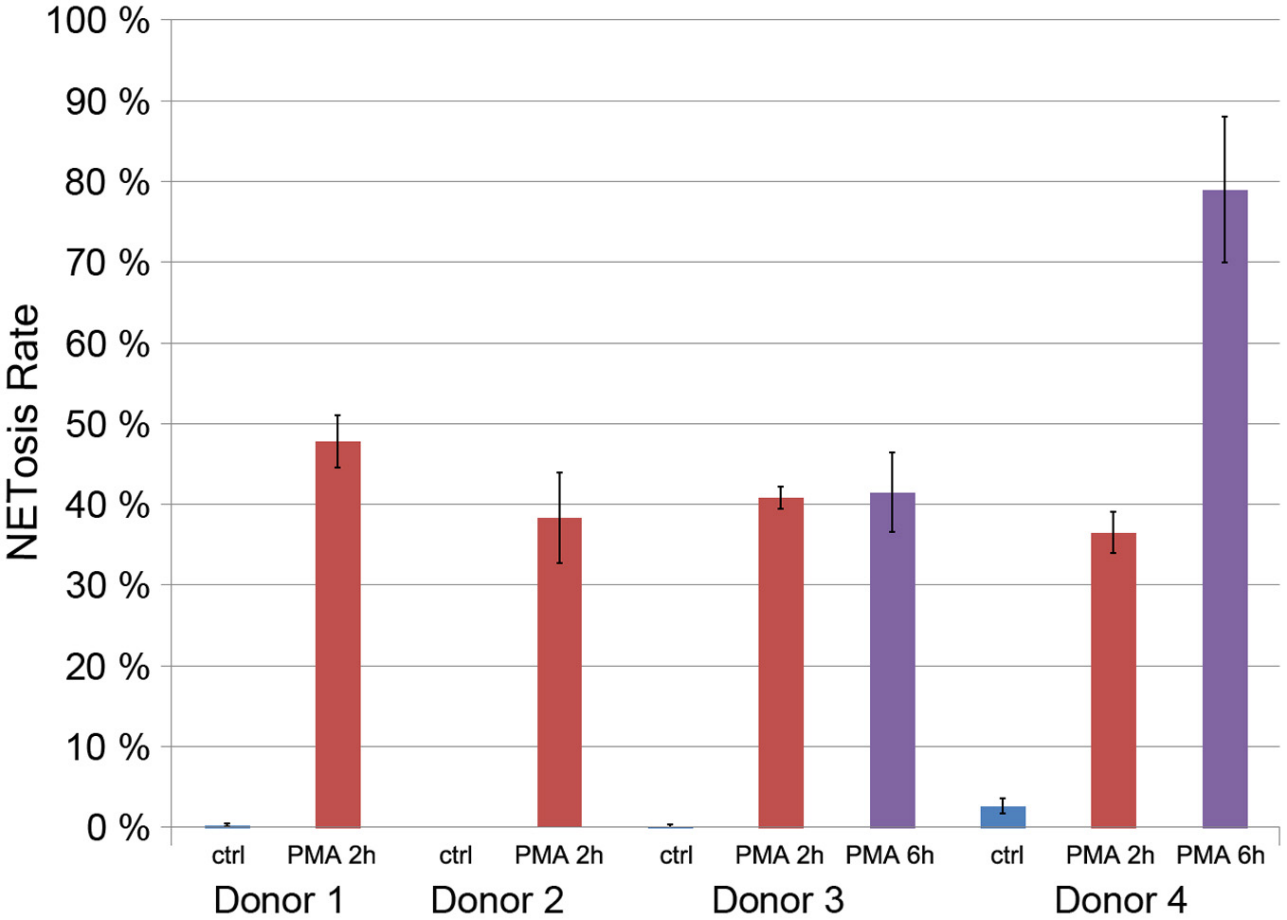
**Quantification of the NETosis rate of neutrophils from for donors unstimulated (blue) and stimulated with 50 nM PMA for 2 h (red) or 6 h (violet, only donors 3 and 4)**.

After 2 h of stimulation with 50 nM PMA, around 40% of the neutrophils isolated from four healthy donors were in the process of NETosis (Figure 4). PMN from two of those donors were stimulated for up to 6 h leading to NET induction in about 80% of neutrophils from one donor (Figure 4). Neutrophils that were cultured without stimulation showed NETosis rates below 5%. These data indicate that there is considerable variation in NETosis rates between donors, especially at later time points.

### Stimulation of pattern recognition receptors

Neutrophils are activated by the presence of pathogens through the detection of pathogen-associated molecular patterns (PAMPs) by pattern-recognition receptors. We tested the ability of different PAMPs to induce NET formation, namely the synthetic lipopeptide (BLP, Pam3Cys-SKKK), a ligand of the TLR1/2-dimer (Aliprantis et al., 1999; Brightbill et al., 1999), a synthetic oligonucleotide rich in CpG-motifs that activates TLR9 (Bauer et al., 2001) and flagellin, a TLR5 ligand (Hayashi et al., 2001).

**Figure 5:**
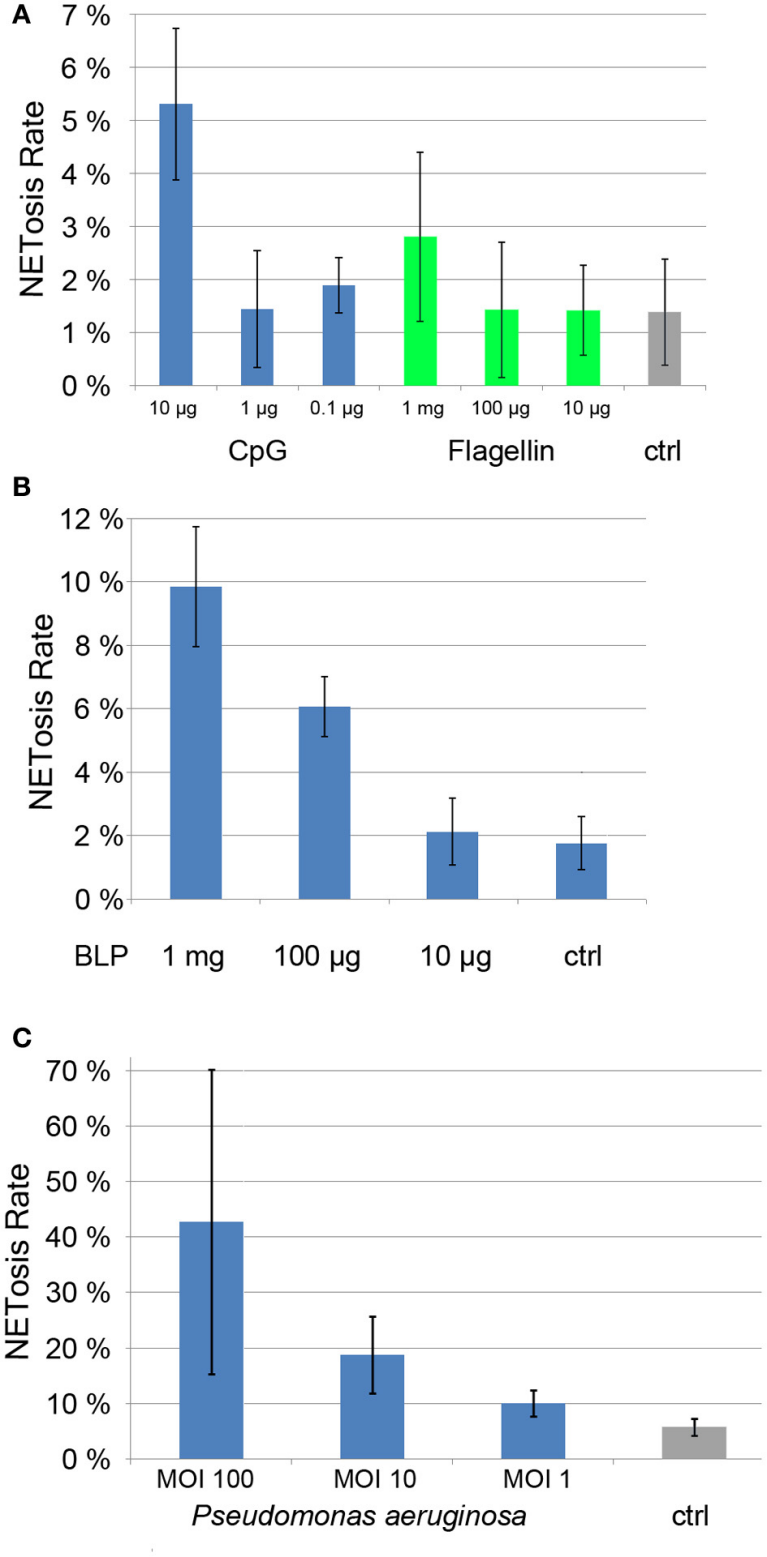
**NETosis rates of ligands of Toll-like receptors (CpG, Flagellin, panel 5A and BLP, panel 5B) as well as NETosis rate of neutrophils cocultured with *Pseudomonas aergunisosa* (panel 5C)**.

Each of these PAMPs induced a dose-dependent degree of NET formation. BLP was active at 1 μg/ml and 100 ng/ml (Figure 5A), while CpG and flagellin induced NETosis at 10 μg/ml and 1 mg/ml, respectively (Figure 5B). While the induction rate of flagellin was rather low (about 3%, Figure 5B), both BLP and CpG induction rates were between 5 and 10% (Figures 5A,B).

Notably, when we cultured PMN with *Pseudomonas aeruginosa* at different multiplicities of infection (MOI) for 5 h, we found a dose-dependent NETosis response. Even a MOI 1 led to clear NET activation of about 10%, while MOI 10 and 100 resulted in NETosis rates of 20 and 40%, respectively. The standard deviation for higher MOIs is high since the amount of bacterial DNA compromises the automatic quantification.

Notably, individual PAMPS are poor NET inducers when compared to bacteria (Figure 5), suggesting that several pattern recognition receptors need to be stimulated in parallel to efficiently drive NETosis.

### NET induced by particles

Monosodium urate crystals (MSU) are present in the joint fluid of patients suffering from gout and are involved in acute inflammatory arthritis. Recently it was found that synovial fluid of gout patients as well as MSU crystals induce NET formation (Mitroulis et al., 2011). Also inorganic particles are known to have inflammatory potential, especially silica particles that when inhaled can lead to severe pulmonary diseases such as chronic obstructive pulmonary disease (Hnizdo and Vallyathan, 2003). Treatment of mouse neutrophils with silica particles resulted in the production of ROS (van Berlo et al., 2010).

**Figure 6:**
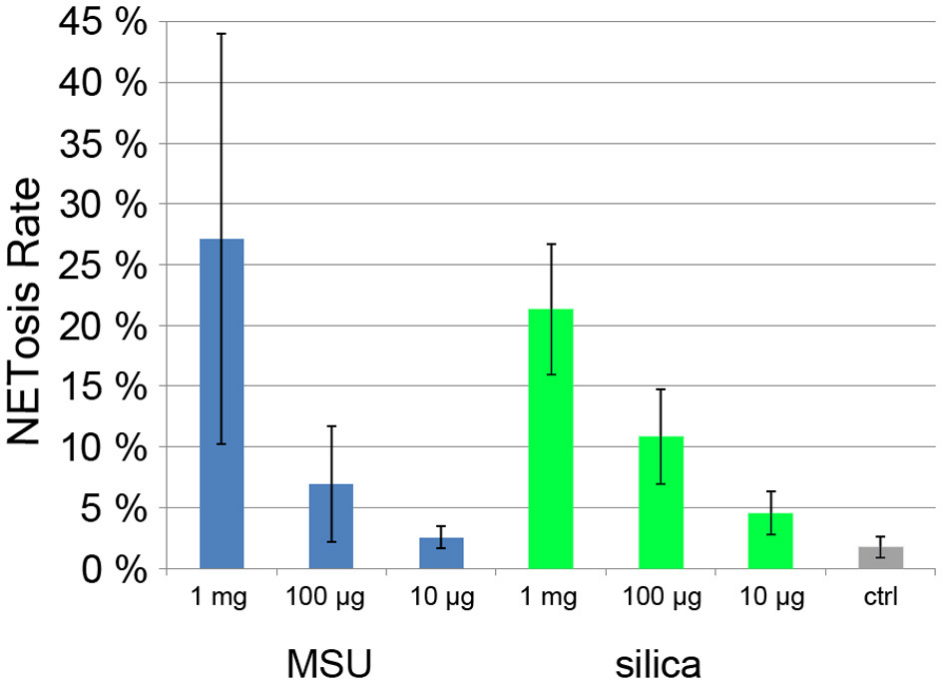
**Quantification of NET induction by monosodium urate (MSU) and silica particles**.

When we incubated human neutrophils with MSU and silica particles, we found a strong, dose dependent NET induction (Figure 6). At 1 mg/ml, NETosis rate was >20% with both types of particle.

### NET induction by cytokine treatment

**Figure 7:**
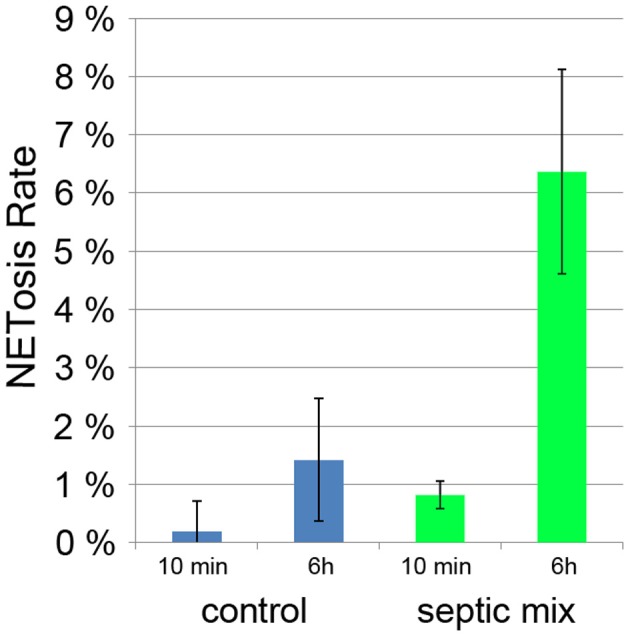
**NETosis rate of neutrophils treated for 10 min and 6 h for with proinflammatory cytokines (TNF-α, G-CSF, and IL-1β)**.

Sepsis is a generalized inflammation potentially leading to multiorgan failure and death. It has been shown that during sepsis, activated platelets can induce neutrophils to produce NETs (Clark et al., 2007). The amount of circulating NETs has been proposed to serve as a prognostic marker with less NETs indicating a better chance of survival (Margraf et al., 2008). Both serum from septic patients and a mixture of inflammatory mediators (TNF-α, G-CSF, and IL-1β) induced NETosis in neutrophils from healthy donors (Kambas et al., 2012). When we incubated neutrophils with this mixture for 6 h, we found a NETosis rate of 6% compared to less than 2% in unstimulated controls (Figure 7). It remains to be determined if neutrophils cultured with inflammatory mediators will produce NETs more readily if treated with a second stimulus.

## Discussion

We present here a method for (semi-) automatic quantification of the induction of Neutrophil Extracellular Traps. The protocol can be used with simple microscopic equipment (fluorescence microscope with digital camera), but it is also suitable for high-throughput screening using micro titer plate format and hardware-based autofocussing. In this case, the chromatin antibody should be used directly labeled to minimize pipetting steps during preparation of the samples.

The method detects the decondensation of chromatin which is a prerequisite of NET formation. We measured a decrease of Hoechst 33342 fluorescence and an increase of chromatin staining already 10 min after PMA stimulation (Figure 2). After about 120 min, plateaus for both fluorescence intensities are reached and remain stable until most cells have reached the final phase of NETosis or have released NETs.

The increase in chromatin fluorescence intensity allows to detect neutrophils even in early phases of NETosis. Setting the threshold above the low chromatin staining of neutrophils which have not (yet) expanded their nuclei, cells with relaxed chromatin can reliably be segmented due to the increase of their fluorescence signal (Figures 1G,H; Figure 3H). Although the intensity of Hoechst 33342 staining decreases over time, both nuclei of resting and stimulated neutrophils can be segmented to allow identification and enumeration of all cells in the micrographs (Figure 3G).

If NETs are produced by several adjacent neutrophils and detected as one fluorescence event, they can possibly be segmented by the computer as one structure (yellow arrowheads in Figure 3I). The number of NETotic events will then be underestimated. To avoid this, a lower number of cells can be seeded, or the cells can be stimulated for a shorter period of time to avoid formation of confluent NETs. The optimal time point for quantification can be determined in a time course experiment.

This quantification method provides results with low standard deviation under conditions with low and intermediate NETosis rate (Figures 5A,B; Figure 7). Conditions that induce higher PMN mobility (MSU crystals, Figure 6) or a second source of DNA (i.e., cocultivation h pathogens, Figure 5C) result in a higher standard deviation since the number of cells per micrograph will have great variations, and the pathogen DNA will hamper proper Hoechst 33342 signal segmentation. Additionally, these conditions can result in a higher rate of cells that detach from the plate and will be lost during washing steps. In this case, lower standard deviation can be achieved by fine-tuning the cell number and the duration of the experiment.

The protocol presented here might prove useful for the detection of molecules which can regulate the signaling cascade that lead to NET formation and could thus be used as therapeuticals.

### Conflict of interest statement

The authors declare that the research was conducted in the absence of any commercial or financial relationships that could be construed as a potential conflict of interest.
